# Influence of Air Mass Source Regions on Signatures of Surface-Active Organic Molecules in Size Resolved Atmospheric Aerosol Particles

**DOI:** 10.1021/acsearthspacechem.3c00161

**Published:** 2023-08-02

**Authors:** Tret C. Burdette, Rachel L. Bramblett, Kathryn Zimmermann, Amanda A. Frossard

**Affiliations:** † Department of Chemistry, University of Georgia, Athens, Georgia 30606, United States; ‡ Department of Chemistry, Georgia Gwinnett College, Lawrenceville, Georgia 30043, United States

**Keywords:** Atmospheric Aerosol Particles, Mass Spectrometry, Surfactants, Particle Composition

## Abstract

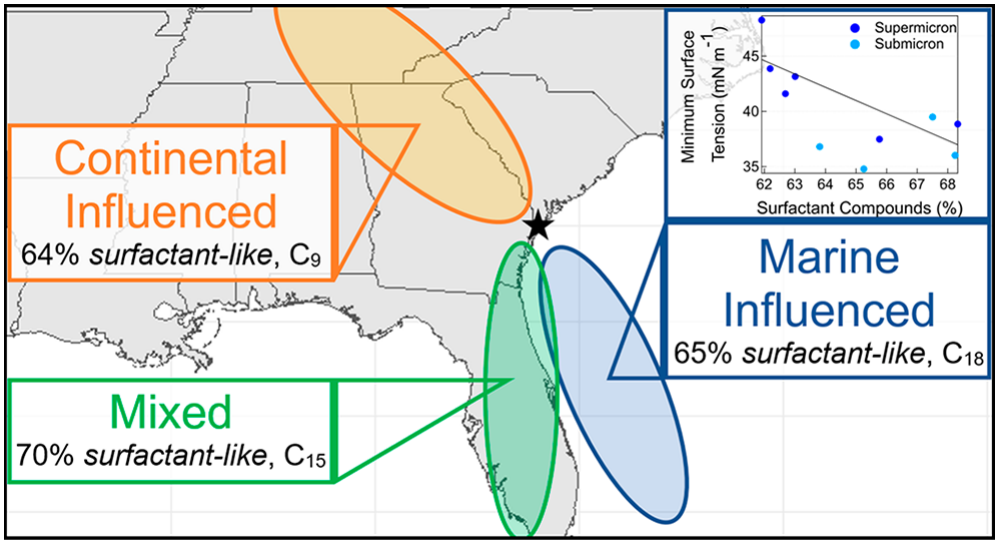

The physical and chemical properties of atmospheric aerosol particles depend on their sources and lifetime in the atmosphere. In coastal regions, sources may include influences from marine, continental, anthropogenic, and natural emissions. In this study, particles in ten diameter-size ranges were collected, and particle number size distributions were measured, at Skidaway Island, GA in May and June 2018. Based on air mass back trajectories and concentrations of major ions in the particles, the air mass source regions were identified as Marine Influenced, Mixed, and Continental Influenced. Organic molecules were extracted from the particles using solid-phase extraction and characterized using tensiometry and high-resolution mass spectrometry. The presence of surfactants was confirmed in the extracts through the observation of significant surface tension depressions. The organic formulas contained high hydrogen-to-carbon (H/C) and low oxygen-to-carbon (O/C) ratios, similar to surfactants and lipid-like molecules. In the Marine Influenced particles, the fraction of formulas identified as surfactant-like was negatively correlated with minimum surface tensions; as the surfactant fraction increased, the surface tension decreased. Analyses of fatty acid compounds demonstrated that organic compounds extracted from the Marine Influenced particles had the highest carbon numbers (18), compared to those of the Mixed (15) and Continental Influenced (9) particles. This suggests that the fatty acids in the Continental Influenced particles may have been more aged in the atmosphere and undergone fragmentation. This is one of the first studies to measure the chemical and physical properties of surfactants in size-resolved particles from different air mass source regions.

## 1.0 Introduction

The chemical composition of aerosol particles affects their ability to act as cloud condensation nuclei and form cloud droplets.^1−3^ The sources of particles can influence their composition and physical properties,^4,5^ thus modulating their direct and indirect influence on the climate. Understanding the chemical composition and physical properties of particles is important for accurately modeling both the impacts of the direct and indirect effects of particles on the current and future climate.^6^

While coastal regions may be influenced by air masses originating over the ocean, this is often not the only source of particles in these regions. Atmospheric aerosol particles in coastal regions consist of contributions from multiple sources, including marine, natural terrestrial, and anthropogenic sources.

Marine aerosol particles can be both primary and secondary in origin and vary in composition, accordingly. Primary marine aerosol particles are generated through bubble bursting at the sea surface.^7^ These particles are comprised of sea salt, with high concentrations of sodium and chloride, and ocean-derived organic compounds.^8^ The organic fraction of marine aerosol particles increases as particle size decreases and is more significant in submicron particles.^9,10^ The fraction of organic mass can change, depending on the biological activity of the water from which the particles are produced.^8^ Marine aerosol particles can be transported long distances and are often measured in high concentrations in coastal environments.^11−13^ Immediately upon emission, primary marine aerosol particles can participate in photochemical reactions, resulting in the fragmentation of large compounds to create volatile organic compounds (VOCs) and/or change chemical properties of the particles.^14−16^ Secondary marine aerosol particles are formed through the oxidation of gases emitted from the sea surface, as well as the oxidation of VOCs from primary marine aerosol particles.^17,18^ Non-sea salt sulfate can also be formed through the oxidation of dimethyl sulfide (DMS), emitted from the ocean, and contribute to a larger fraction of sulfate in marine aerosol.^19,20^

Coastal and estuarine plants can emit VOCs that form organic acids, including formate and acetate.^21−23^ Another major natural, terrestrial source of particles is mineral and soil dust, which can be identified through the presence of calcium and carbonate.^24−26^ The fraction of calcium in dust particles is much larger than that observed in sea spray particles.^26−28^ Dust has been observed as a major contributor to particle concentrations along the east coast of the United States.^23,29,30^

Anthropogenic aerosol particles can originate from a variety of sources and, thus, have diverse and complex compositions. Combustion emissions from biomass burning and fuel emissions have been linked to multiple inorganic ions, including sulfate, nitrate, and potassium in urban particles.^31^ Sulfate can also be produced through industrial methods and power generation.^23,32^

The different sources of aerosol particles, both natural and anthropogenic, result in a complex mixture of chemical and physical properties.^33^ High-resolution mass spectrometry provides insight into the complex mixture of particles through analysis of individual chemical formulas that can be categorized by hydrogen-to-carbon (H/C) and oxygen-to-carbon (O/C) ratios.^34−38^ This allows for broad classifications of organic molecules into different groups, such as aromatic compounds or fatty acids. Fatty acids are particularly of interest because of their surface-active properties and ability to act as surfactants.^35,39^ Fatty acids can originate from a variety of natural and anthropogenic sources, and their carbon chain lengths can vary, depending on sources and atmospheric processing.^40^

Surfactants are organic molecules that reduce the surface tension at the interface of a solution.^41,42^ Surfactants have been measured in seawater^43−46^ and in marine aerosol particles.^47,48^ Other studies have observed surfactants in anthropogenic-influenced particles,^49,50^ ambient urban particles,^51^ and biogenic terrestrial particles.^39,52^ Surfactants can impact particle hygroscopic growth^53,54^ and their potential to act as cloud condensation nuclei.^55−57^ Recent work has used high-resolution mass spectrometry to identify fatty acids and other surfactants in aerosol particles.^34,35^ These results can be combined with surface tension measurements to confirm the presence of surfactants.^58^

In this study, high-resolution mass spectrometry was used to identify and characterize surfactants extracted from size-separated aerosol particles collected from three different air mass source regions sampled from Skidaway Island, GA in May and June 2018. Particle number size distributions were collected simultaneously and varied in number concentrations, depending on the air mass source region. Inorganic ions measured with ion chromatography and air mass back trajectories confirmed three different air mass source regions. Here, the presence of surfactants in these particles was confirmed using tensiometry, chemical formulas were identified as surfactant-like using high-resolution mass spectrometry, and the chain lengths of fatty acids are reported for the samples from the different air mass source regions. The goal of this work is to determine the mass spectral signatures and sources of surfactants in size-resolved coastal aerosol particles and demonstrate the presence of surfactants in coastal aerosol particles from both marine and nonmarine sources.

## 2.0 Method

### 2.1 Particle Sample Collection

Aerosol particle samples were collected at Skidaway Institute of Oceanography (31.989584° N, 81.021622° W) outside of the Geochemistry Building at a height of ∼1.5 m. The sampling site is ∼35 m from the Skidaway River, ∼7 km from Wassaw Sound, and ∼11 km from the outer coast. Sampling was done over a two-week period from May 23, 2018 to June 5, 2018. However, there was no sample collection on May 27, 2018 due to the weather conditions, which included significant rainfall.

Size-fractionated particle samples were collected using two 8-stage Micro-Orifice Uniform Deposition Impactors without rotators (MOUDI, Model 100-NR, TSI, USA).^59^ Particles were collected on precombusted aluminum substrates for each stage of the MOUDI and a precombusted quartz fiber filter after the final stage. Samples were collected for 20–24 h at a flow rate of 30.0 L min^–1^. Incoming air was dried by using a diffusion dryer prior to sampling. After sample collection, the substrates were folded and placed in precleaned, individual 2-dram glass vials, wrapped in aluminum foil, and frozen until further analysis at the University of Georgia.

Three MOUDI sample time periods, including the samples from each of the 10 size fractions, were selected for analyses in this study. These samples were collected on May 26–27 (5/26/18 9:37 to 5/27/18 9:33), May 28–29 (5/28/18 10:44 to 5/29/18 9:50), and June 4–5 (6/4/18 10:24 to 6/5/18 7:20). As discussed in Section 3.0, these three time periods represent meteorological variability over the entire project. Each of the three sample time periods contains 10 individual samples, one in each particle size fraction (*n* = 30 total).

Field blanks were collected for the MOUDI samples on May 25 (5/25/18 9:32), May 28 (5/28/18 10:02), and June 3 (6/3/18 10:18). Substrates were loaded in the MOUDI, and it was connected in line for 10 s, without flow. The blanks from May 28 to June 3 were used in this study.

### 2.2 Particle Number Size Distributions

Particle number size distributions were measured using an aerodynamic particle sizer (APS, Model 3321, TSI, USA) for particles with aerodynamic diameters ranging from 0.5 to 20 μm in diameter, operating with scan times of 1 min. Submicron particle number size distributions were measured using a scanning electrical mobility spectrometer (SEMS, Model 2100, Brechtel, USA) for particles with mobility diameters ranging from 10 nm to 1 μm, operating with scan times of 50 s. The APS and SEMS instruments shared a single inlet containing an upstream diffusion drier, and the sample flow was split using a custom-made stainless-steel splitter. The silica gel in the dryer was changed daily to maintain a sample flow relative humidity of <70%.

The particle mobility diameters measured with the SEMS were converted to aerodynamic diameters using an assumed particle density of 2.12 g cm^–1^.^60,61^ The APS and SEMS number size distributions were averaged over the same 10 min intervals and then merged at an aerodynamic particle diameter of 932 nm, similar to previous work.^60−62^ An assumed particle density of 2.12 g cm^–1^ resulted in the best overlap between the SEMS and APS number size distributions for the three sample time periods. The number size distributions were used to calculate the total number and mass concentrations for each 10 min interval, as well as the particle masses in each of the MOUDI diameter ranges. The APS number size distributions for the June 4–5 sample were scaled to correct instrument error for low particle counts (see Text S1 in the Supporting Information).

### 2.3 HYSPLIT Back Trajectories

The Hybrid Single-Particle Lagrangian Integrated Trajectory model (HYSPLIT, NOAA) was used to calculate the back trajectories of the air masses and particles sampled during the campaign.^63^ Model back trajectories were created daily at altitudes of 500 m above the mean sea level and for a duration of 48 h, over the entire project. During individual MOUDI sampling times, 48 h model back trajectories were calculated every 12 h.

### 2.4 Particle and Organic Molecule Extraction

Water extractable species were extracted from the aluminum substrates and quartz fiber filters using a method of sonicating and/or vortexing, as described previously^34,35,60,64−66^ and briefly here. First, vials containing the substrates from the three selected samples were brought to room temperature, 5 mL of ultrapure water was added to each, and the vials were sealed. The substrates from the samples on May 26–27 and May 28–29 were agitated through two alternating cycles of vortexing and sonicating for 5 min each and subsequently transferred to individual precleaned 3-dram vials. This procedure was repeated a second time for these two samples, resulting in a total extract volume of 10 mL for each substrate. The substrates from the June 4–5 sample, with the addition of 5 mL of ultrapure water, were vortexed for 5 min, stored at 4 °C for 15 h, and were then vortexed again for 5 min. The solutions were transferred to individual precleaned 3-dram vials. Then, an additional 5 mL of ultrapure water was added to each substrate vial, followed by 5 min of vortexing and 30 min at rest, resulting in a total extract volume of 10 mL for each substrate. These two methods were assumed to extract organic species with similar efficiencies.

The 10 mL solutions, extracted from the substrates from all three MOUDI samples, were filtered using 0.45 μm poly(ether sulfone) membrane syringe filters into individual precleaned and preweighed 3-dram vials. All of the vials with the extract solutions were then weighed to obtain their exact masses.

The large organic compounds were then concentrated and separated from the rest of the sample matrix using a solid-phase extraction (SPE) method described by Burdette and Frossard.^34^ Briefly, the 10 mL extracts for each sample were processed through two SPE cartridges, ENVI-18 (C18 sorbent material, 0.5 g bed weight, MilliporeSigma, USA) and ENVI-Carb (graphitized carbon sorbent material, 0.5 g bed weight, MilliporeSigma, USA).

First, the ENVI-18 cartridges were conditioned with 6 mL of acetonitrile (MilliporeSigma, USA) and rinsed with 12 mL of ultrapure water. The 10 mL solutions from the initial sample extracts were pipetted into the cartridges and passed through the SPE material without vacuum. The eluates were collected in precleaned vials to save for extraction with the ENVI-Carb cartridge. The ENVI-Carb cartridges were conditioned with 6 mL of acetonitrile (MilliporeSigma, USA) and rinsed with 12 mL of ultrapure water. The 10 mL eluates from the ENVI-18 extractions were pipetted into the ENVI-Carb cartridges and passed through the SPE material without vacuum. The final eluates were collected in precleaned vials for ion chromatography analyses, as described in the next section.

For the elution of the organic molecules from the ENVI-18 and ENVI-Carb cartridges, the cartridges were rinsed using 12 mL of 0.1% triethylamine (MilliporeSigma, USA) in ultrapure water to remove any remaining salt in the SPE sorbent material.^34^ The cartridges were dried using vacuum, and the organic material was eluted in individual precleaned 1-dram vials using 4 mL of acetonitrile. The acetonitrile was evaporated using dry nitrogen gas (99.998% purity, Airgas, USA), and the vials with dried organic extracts were stored at 4 °C until analysis.

### 2.5 Measurements of Major Ions

The concentrations of major ions were measured using two Dionex Integrion high-pressure ion chromatography (HPIC, Thermo Fischer Scientific, USA) instruments. Samples and blanks were prepared by filling ion chromatography vials with 5 mL of the ∼10 mL final eluates from the solid-phase extractions. The autosampler split the volume of each sample between the two HPIC instruments, with one column to quantify the cations (IonPac CS12A, Thermo Fischer Scientific, USA) and the other to quantify the anions (IonPac AS18, Thermo Fischer Scientific, USA). Here, we measured the concentrations of cations lithium, sodium, ammonium, potassium, magnesium, and calcium and anions fluoride, acetate, formate, chloride, nitrite, carbonate, sulfate, and nitrate in each solution. The concentrations of the corresponding sample blanks were subtracted from the samples. Concentrations in solution were calculated from calibration curves for each ion of interest. Ion concentrations in particle samples were derived using the total volume of air sampled.

### 2.6 Surface Tension Measurements

The eluted and dried organic extracts from the ENVI-18 cartridges were rehydrated for surface tension analysis. Room-temperature organic extracts were dissolved in 40 μL of ultrapure water, and the surface tensions were measured using a pendent drop tensiometer (OCA 15EC, DataPhysics, Germany). The 40 μL solutions were suspended from syringes with 0.30 mm diameter needle tips for ∼30 s, at which point the surface tension was then measured continuously for ∼10 s to determine an average droplet surface tension. This process was repeated to obtain triplicate droplet measurements for each sample. The surface tension of the 40 μL hydrated extract is referred to herein as the surface tension minimum.^34,43,58,65,66^ Surface tension minimums are reported for the May 26–27 and May 28–29 samples. Surface tension minimums were not measured for the June 4–5 sample.

### 2.7 High-Resolution Mass Spectrometry

Organic fraction eluates were further hydrated and combined for analysis using high-resolution mass spectrometry. The dry ENVI-Carb extracts were rehydrated to 500 μL with a 1:1 mixture of methanol (ACS grade, Thermo Fisher Scientific, USA) and ultrapure water, followed by 5 min of sonicating and 30 s of vortexing. Then, the 500 μL the ENVI-Carb rehydrated extracts were transferred to the vials containing the 40 μL of hydrated ENVI-18 extracts for the corresponding samples. The combined extracts were then hydrated to final volumes of 1 mL, and an internal reference standard, Genistein (MilliporeSigma, USA), was added to a final concentration of 5 μM.

The combined, rehydrated samples and blanks were analyzed at the Proteomics and Mass Spectrometry Facility (PAMS) at the University of Georgia using an electrospray ionization quadrupole time-of-flight mass spectrometer (ESI-Q-TOF-MS; Impact II, Bruker, USA). This instrument has high mass accuracy and high resolution (∼20,000 resolving power at 400 Da), and all analyses were done using the negative ionization mode. A loop injection was used to introduce the blanks and samples to the mass spectrometer, and methanol was used as the carrier at a flow rate of 180 μL h^–1^. For each sample, an instrument run was started, where a blank corresponding to the selected sample was injected. Once the blank was finished, the sample was injected in the same run. After the sample was collected, a standard calibration mixture was injected. This process was repeated for each sample, and the instrument acquired mass spectra across a mass-to-charge (*m*/*z*) range of 50–1500 Da.

The mass spectra were initially processed using the instrument software (DataAnalysis, Bruker). The calibration was applied, and the mass accuracy of the Genistein was validated to ensure that the error was within 0.5 ppm. The mass spectral data was exported, and a custom MATLAB code was used to assign chemical formulas to the negative ion peaks in each sample spectrum, with a mass tolerance of 1.0 ppm.^38,58^ Chemical formulas were assigned with elemental ranges of C_0–50_, H_0–100_, O_0–30_, N_0–6_, and S_0–2_, following elemental constraints described by Stubbins et al.^67^ If multiple formulas were assigned to an individual peak, further analyses, including the Kendrick mass defect analysis, were used to determine a single, unambiguous formula.^38,68,69^ Only formulas that were unambiguously assigned to a specific peak were used in the analysis of the mass spectral data in this study. Formulas that contained both high O/C (oxygen to carbon, >0.6) and low H/C (hydrogen to carbon, <0.7) ratios were removed, since they were unlikely to be real organic formulas.^38,67^

### 2.8 Data Analyses and Statistical Tests

The programming language R was used for statistical analyses of aerosol particle chemical and physical properties as well as mass spectral data. Comparisons between sets of data were done using unpaired *t*-tests, and correlations were determined using Pearson correlation coefficient (*R*). Herein, any test with a *p*-value of <0.05 is labeled as “significant”.

## 3.0 Results and Discussion

### 3.1 Classification of Air Mass Source Regions

**Figure 1:**
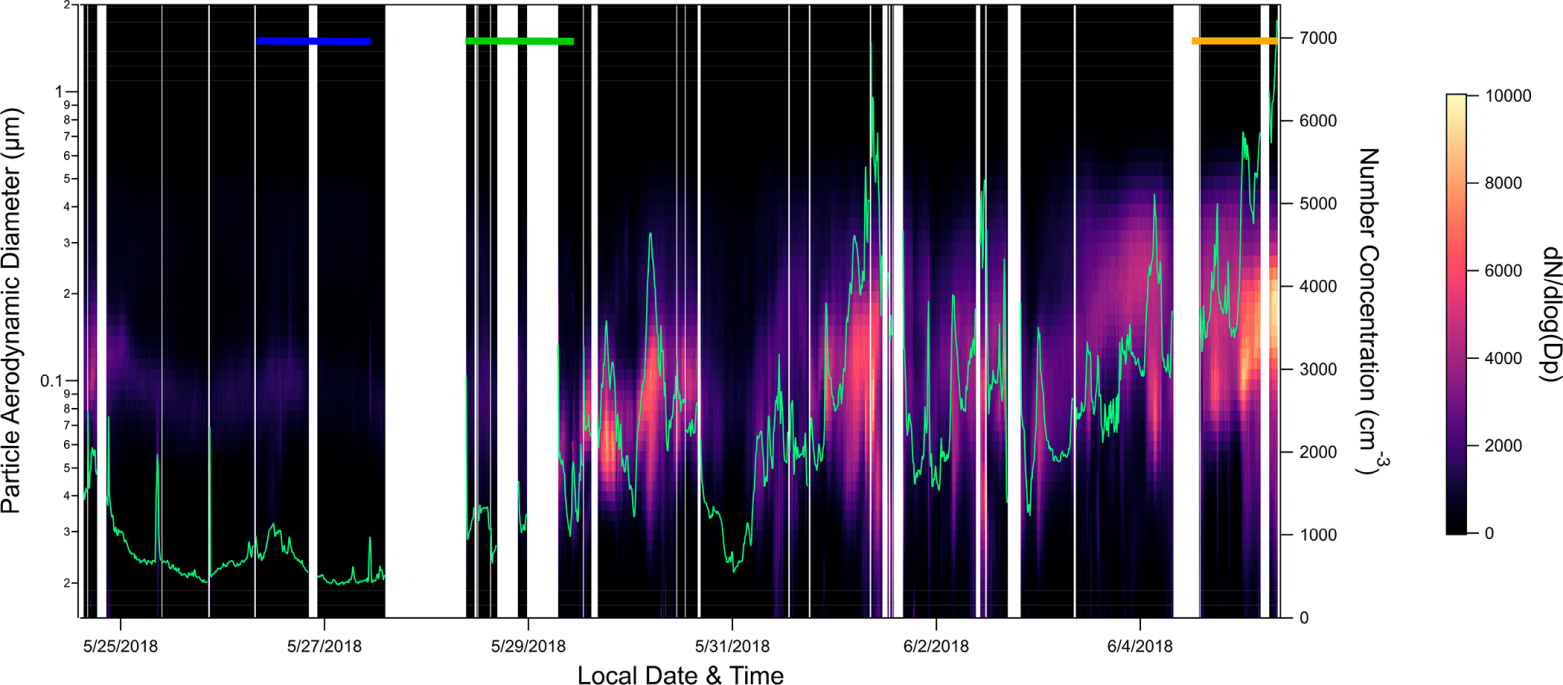
Number size distributions and total particle number concentrations (green) across the full sampling time for particles < 2 μm in diameter. White spaces indicate missing data or periods of instrument maintenance. Horizontal lines at the top indicate the three sampling periods investigated in this study, including Marine Influenced (blue), Mixed (green), and Continental Influenced (orange).

During the two-week sampling period, particle number concentrations increased with time (Figure 1). This general increase in number concentration corresponds to a shift in the local wind direction, which corresponded to changes in the origins and back trajectories of air masses that were sampled (Figures S1 and S2 in the Supporting Information). At the start of the sampling period, during the time frame of May 24–28, the back trajectories demonstrate that the air masses originated from southeast of the sampling site, over the Atlantic Ocean (Figure S1). This sampling period and the MOUDI sample collected therein (May 26–27) are defined as “Marine Influenced”, based on the air mass back trajectories originating from over the ocean and spending more than 75% of the time over the ocean prior to sampling (Figure S3 in the Supporting Information).^16^ However, this sampling period is not purely “clean marine” and may have additional influences from atmospheric aging and mixing with other sources.^16^ On May 28, 2018, the wind direction shifted, and the back trajectories show that the air masses originated from over Florida and from southwest of the sampling site, over the Gulf of Mexico (Figures S1 and S2 in the Supporting Information). This sampling period and the MOUDI sample collected therein (May 28–29), are defined as “Mixed” due to the overall mixture of air mass source regions (Figure S4 in the Supporting Information). On June 2, 2018, the back trajectories indicate that the air masses were traveling across the continental United States, originating northwest of the sampling site (Figure S2 in the Supporting Information). This sampling period and the MOUDI sample collected therein (June 4–5), are defined as “Continental Influenced”, since the air masses all originated from over the continental United States and may include a mixture of different continental sources, both natural and anthropogenic (Figure S5 in the Supporting Information).

**Figure 2:**
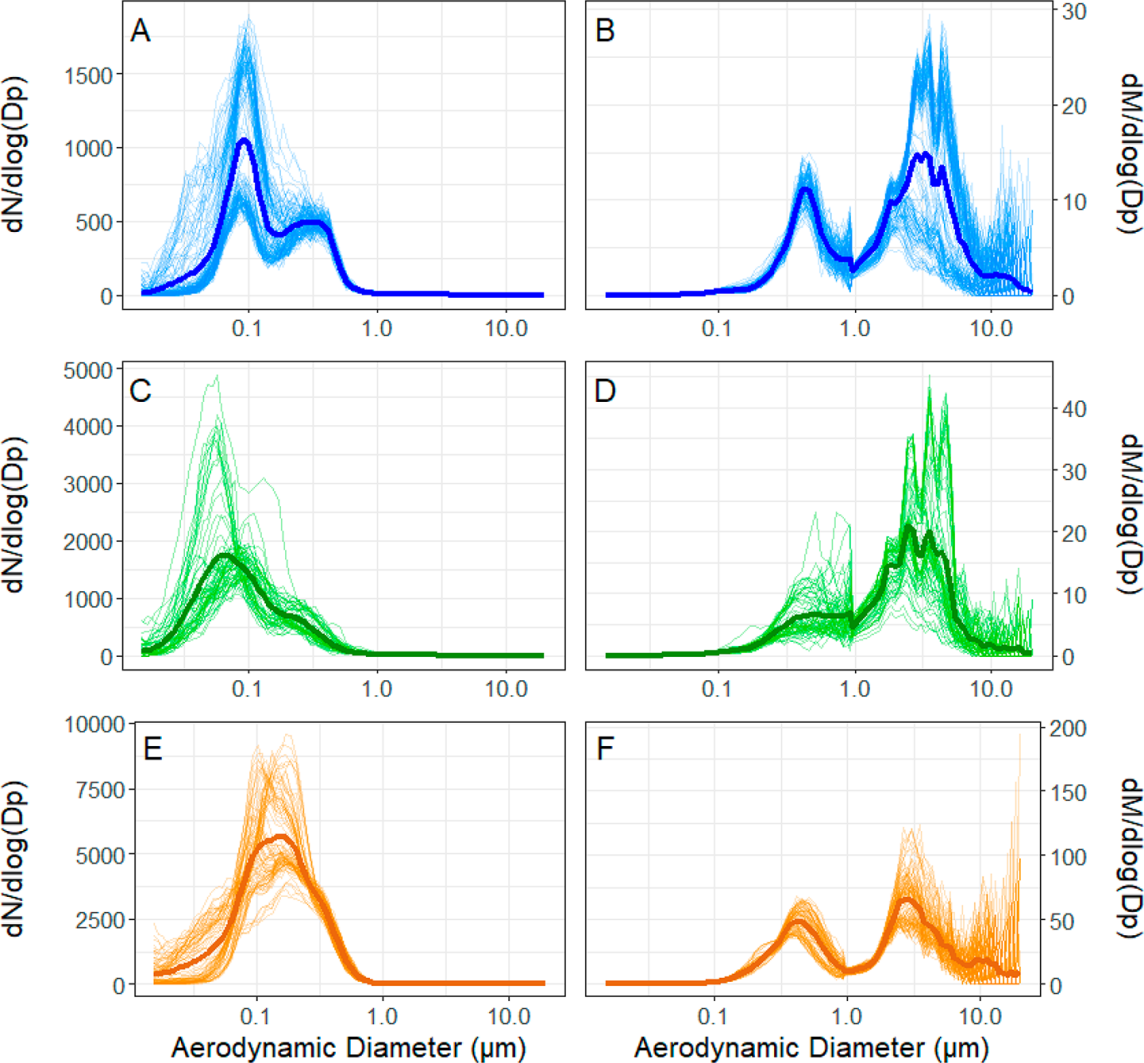
(A, C, and E) Number and (B, D, and F) mass size distributions measured for the Marine Influenced samples (panels A and B), Mixed samples (panels C and D), and Continental Influenced samples (panels E and F). Thin lines represent individual 10 min averages (Marine Influenced: *n* = 132; Mixed: *n* = 61; and Continental Influenced: *n* = 92), and darker lines represent the average for the sampling period, for both the number and mass size distributions. Note that the *y*-axis ranges vary.

The number size distributions for the three selected MOUDI sample periods were isolated to compare their particle number and mass concentrations (Figure 2), with varying numbers of 10-min average particle number size distributions (132, 61, and 92 for the Marine Influenced, Mixed, and Continental Influenced sample periods, respectively). Marine Influenced particles have the lowest average d*N*/d(log *D*_p_), compared to the other two sampling periods. Additionally, Marine Influenced particles show a bimodal distribution in the number size distributions, with the Aitken mode at 93 nm diameter and the accumulation mode at 272 nm, respectively (Figure 2). This bimodal peak shape in the number size distribution is unique to the Marine Influenced particles and is consistent with number size distributions of marine aerosol particles measured in previous studies.^70-72^ Similar distributions with Aitken and accumulation modes observed in marine aerosol particles have been attributed to non-precipitation cloud cycle processing.^72,73^

The number size distributions from the Mixed particles have a smaller average peak diameter of 62 nm as well as an overall number concentration higher than that of the Marine Influenced samples (Figure 2). The higher particle number concentration also corresponds to a higher particle mass during the Mixed sampling period compared to the Marine Influenced. Additionally, the Mixed number size distributions have a shoulder around 224 nm diameter but do not have the full second peak observed in the Marine Influenced (Figure 2). Single modes in number concentrations were previously observed in anthropogenic and continental influenced aerosol particles,^71,72^ consistent with the single modes observed here in the Mixed and Continental Influenced particle number size distributions.

The particle number concentrations were significantly higher in the Continental Influenced particles compared to the other two sampling periods (Figure 2). These number size distributions also had a larger diameter mode at ∼155 nm (Figure 2). The higher particle number concentration and larger average particle size contributed to a higher mass concentration for this sampling period (Figure 2).

The different shapes of particle number size distributions, as well as their peak locations, add confirmation that these three air masses were distinct from each other, resulting in different sources and probable variability in the fractions of primary and secondary (or chemically aged) particles in each population. The lower number concentrations and the distinct double peak in the number size distribution of the Marine Influenced sample indicates that these particles were primarily marine particles and had minor anthropogenic influences.^71,72^ The Continental Influenced number size distributions show a clear shift in the particle sizes to larger bins. This and the overall higher number concentrations may indicate particle growth in the atmosphere, contributing to the accumulation mode.^71^ Alternatively, the larger particle sizes could indicate the presence of dust from long-range transport.^74^

### 3.2 Major Ion Concentrations Vary with Particle Size and Source Regions

**Figure 3:**
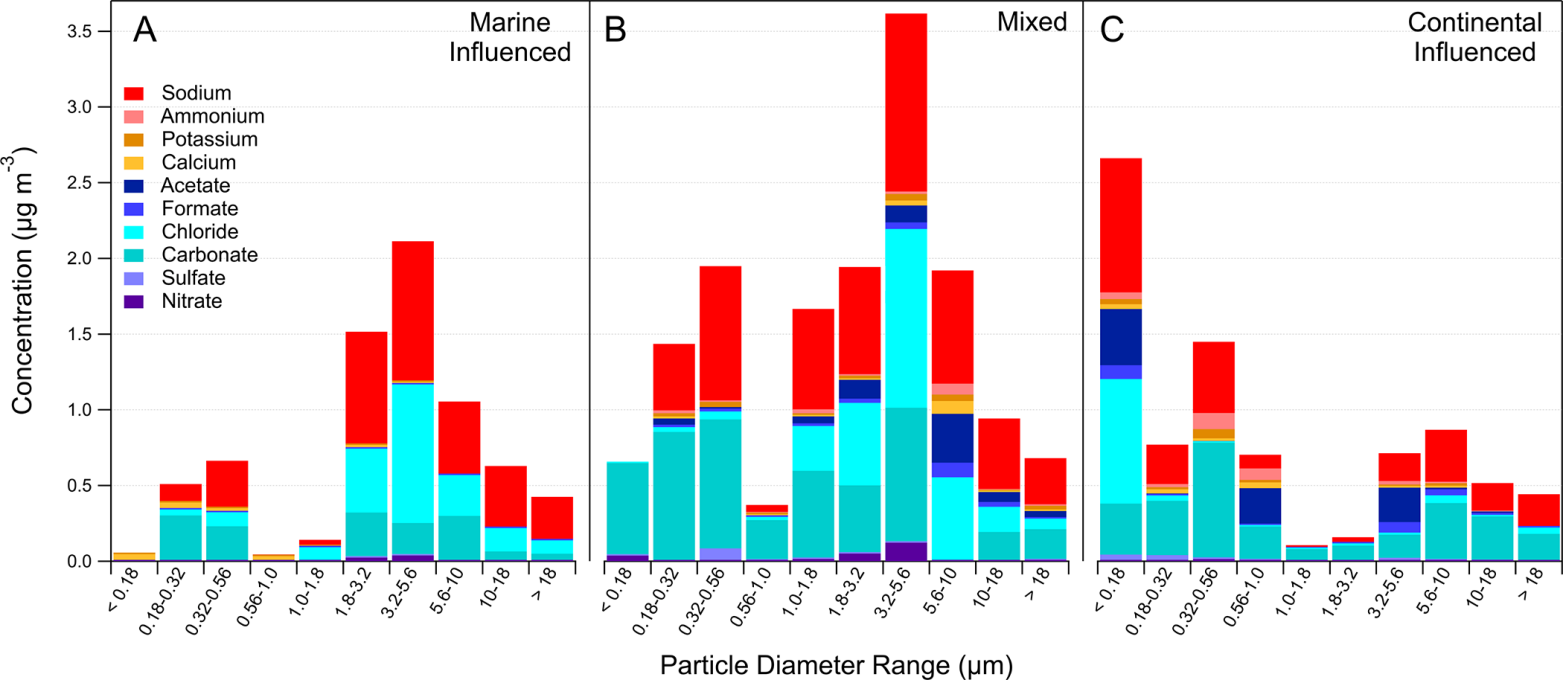
Distribution of ion concentrations in the 10 different size bins collected for each sample, including (A) Marine Influenced, (B) Mixed, and (C) Continental Influenced air mass source regions. Ions with concentrations <0.02 μg m^–3^ were not included.

The differences in the particle number and mass distributions also correspond to variations in the major ion concentrations from the filters collected in each MOUDI diameter range (Figure 3 and Table S1 in the Supporting Information). HPIC has been used in prior research to identify and quantify the inorganic fraction of aerosol particles and infer particle sources.^23,32,75,76^

The major inorganic ions identified in the Marine Influenced particles were sodium, chloride, and carbonate (Figure 3a, *n* = 10). Sodium and chloride are commonly indicative of sea spray aerosol particles.^23,77,78^ The average fraction of chloride was the highest in the Marine Influenced particles compared to the Mixed and Continental Influenced particles. The ratio of mean sodium to mean chloride in the Marine Influenced particles is 1.6 (*n* = 10), and when compared to the ratio of sodium to chloride in seawater (0.56), demonstrates that these particles could be aged sea spray aerosol instead of freshly emitted.^16,78^ Atmospheric aging of sea spray aerosol particles causes a decrease in the fraction of chloride, and it is commonly replaced with non-sea salt sulfate^78^ or nitrate.^79^ However, in this study, sulfate was measured at low concentrations, around the detection limit, similar to those measured in the field blanks in the Marine Influenced samples. Additionally, the higher ratio of sodium to chloride in these samples also demonstrates that, while these samples are associated with air mass back trajectories from over the open ocean, they are not considered purely clean marine. While the source of carbonate in these samples has not been definitively identified, it is hypothesized that the small mass collected was slightly influenced by minor external mixing with a source of carbonate (such as soils and/or dust). The carbonate in the supermicron particles could also indicate a contribution of African dust, based on the path of the air mass back trajectories.^74^ Most of the measured ion mass is concentrated in the supermicron particles (Figure 3a) and is consistent with the calculated mass distributions (Figure 2b). The inorganic fraction accounted for ∼56% of the total mass calculated from the number size distributions, not taking into account potential sampling losses in the larger size fractions.

The Mixed particles also had high sodium, chloride, and carbonate concentrations, which varied across the different particle diameter ranges (Figure 3b, *n* = 10). The total mass concentrations of the major ions across all diameter ranges in this sample are higher than those of the Marine Influenced samples and include low concentrations of potassium and sulfate that were not detected in the Marine Influenced samples. These low concentrations of potassium and sulfate could indicate a minor influence of aged biomass burning and further demonstrate the mixed nature of this sample.^80,81^ While carbonate was present in some of the Marine Influenced samples, it is present in much larger concentrations in the Mixed particles (Figure 3). Carbonate is often indicative of mineral dust^25,26^ but can also be found in lake spray aerosol particles^82^ and Georgia estuarine water.^83−85^ Carbonate can also originate form cement dust at construction sites.^86^ The largest relative fraction of carbonate is in the submicron particles (Figure 3b). There is also a small fraction of calcium in these particles, which could also be another indicator of mineral dust.^24,25,87^

The supermicron particles in the Mixed sample contained chloride, but the ratio of mean sodium to mean chloride in these particles (1.87) is higher than that of the Marine Influenced samples. This could be attributed to the collection of more coastal and terrestrial aerosol particles, as well as more aged particles.^78,88,89^ This could also suggest an additional source of chloride, such as biomass burning.^90^ The supermicron particles also contained fractions of acetate and formate (up to 13% and 4%, respectively, in the 5.6–10 μm diameter particles). The presence of organic ions including acetate and formate could indicate the terrestrial sources of these particles.^22^ The inorganic fraction accounted for ∼30% of the total mass calculated from the number size distributions, not taking into account potential sampling losses in the larger size fractions.

The Continental Influenced sample had high concentrations of sodium, acetate, and carbonate (Figure 3c, *n* = 10). Compared to the other samples, these particles had the lowest overall sodium and chloride concentrations, which was expected since the air mass back trajectories did not originate from over the ocean (Figure 3). The concentration of sodium is similar to that of carbonate across most of the particle size ranges, suggesting that both might come from similar mineral dust sources^91−93^ or contain coagulated dust and sea spray particles due to processing in the atmosphere. This sample had the highest overall concentrations of ammonium and sulfate, observed in the submicron particles, which could be associated with industry and power generation.^23,32^ Additionally, the Continental Influenced sample had the largest overall fraction of the organic ion acetate, which may be associated with other terrestrial sources, such as vegetation and soil.^94,95^ This sample also had the highest overall mass concentration calculated from the number size distributions (Figure 2f) but not the highest concentration of major ions. The total mass of the major ions, including the inorganic fraction and the measured organic acids, accounts for ∼31% of the total calculated mass from the size distributions, excluding any sampling losses in the larger size fractions. This indicates that a significant fraction of the collected mass is likely organic matter, some of which will be discussed in the next section.

The Marine Influenced particles have more mass in the supermicron particles, while the Mixed particles have a similar distribution of masses in submicron and supermicron particles. The Continental Influenced particles have more mass in the submicron particles, compared to the supermicron particles. Together, the HYSPLIT analyses, the variation in the particle mass distributions, and the varying ion distributions confirm the three distinct sources of particles sampled during the Marine Influenced, Mixed, and Continental Influenced time periods.

### 3.3 Mass Spectral Comparison of Organic Fraction of Particles

The extracted organic fraction for each of the particle samples in each size fraction was analyzed using high-resolution mass spectrometry, and organic formulas were identified. In total, ∼7000 unique formulas were identified across the three particle sample periods (*n* = 30). The Marine Influenced samples had the highest total number of organic formulas identified (∼5000), followed by the Continental Influenced (∼3000) and Mixed (∼2000) samples.

**Figure 4:**
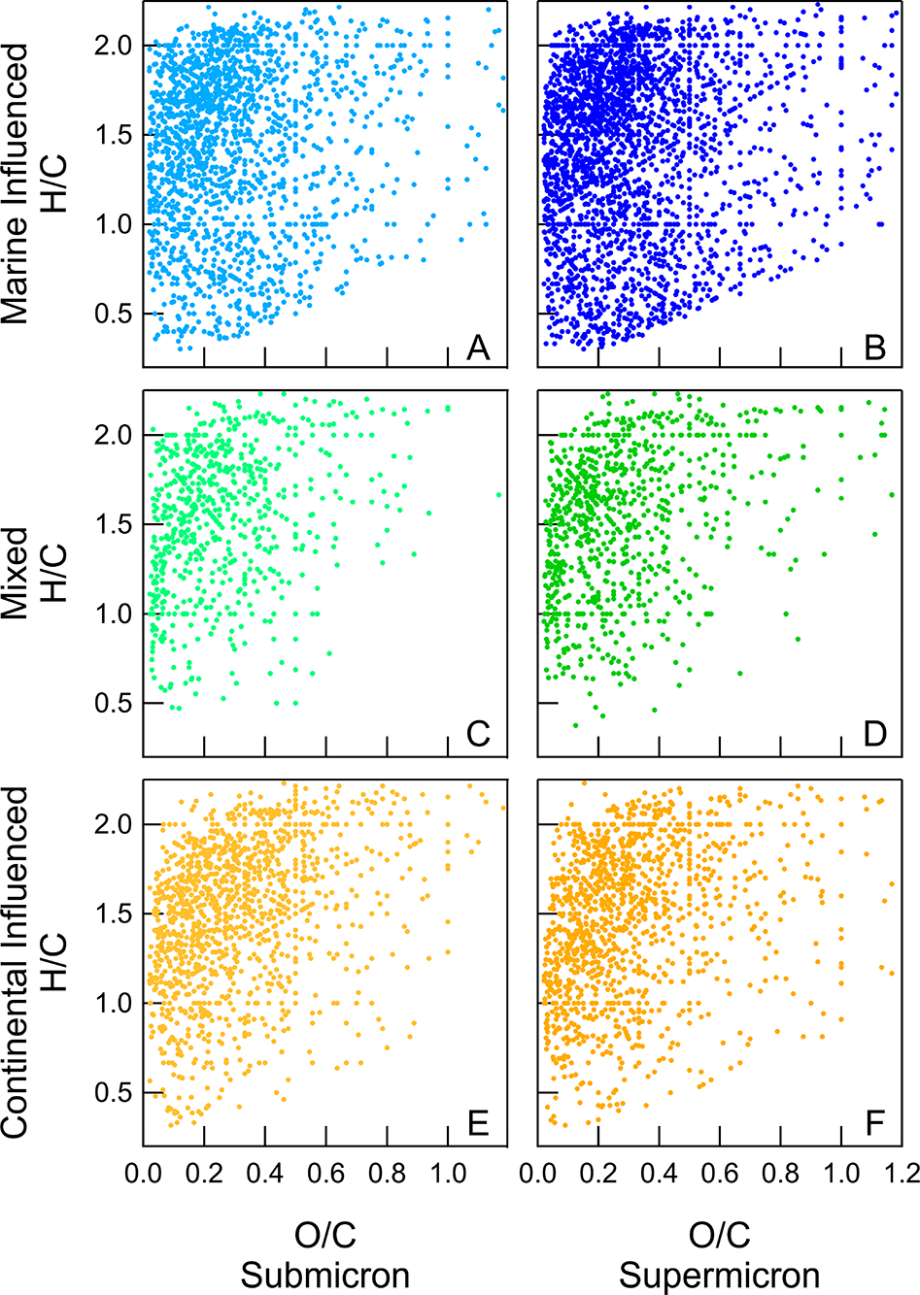
Van Krevelen diagrams showing H/C and O/C for the organic formulas identified in the organic fraction of particles sampled from the Marine Influenced (A and B), Mixed (C and D), and Continental Influenced (E and F) samples. The organic formulas from the mass spectral data are separated by submicron (panels A, C, and E) and supermicron (panels B, D, and F) particle sizes.

The van Krevelen diagrams (Figure 4) show the relative H/C and O/C ratios of each chemical formula identified in the different samples, in the submicron and supermicron size ranges. The majority of formulas identified in the three samples, in both size ranges, have low O/C and high H/C values (Figure 4). The average values of H/C and O/C ratios were similar for each of the size bins across all three samples (Table 1), with generally high H/C (∼1.50) and low O/C (∼0.3), indicative of hydrocarbon-like molecules, as discussed later.

**Table 1:** H/C, O/C, Percent Aliphatic (% Aliph.), Percent Aromatic (% Arom.), Percent Lipid-Like (% Lipid-Like), Percent Secondary Organic Aerosol Like (% SOA-Like), Percent Surfactant-Like (% Surf-Like), and Minimum Surface Tension (ST Min.) Measured for Organics Extracted from Particles in the Three Air Mass Source Regions, as a Function of Particle Diameter Range and for the Average (Avg.) of the Submicron and Supermicron Particle Diameters

	<0.18 μm	0.18–0.32 μm	0.32–0.56 μm	0.56–1.0 μm	1.0–1.8 μm	1.8–3.2 μm	3.2–5.6 μm	5.6–10 μm	10–18 μm	>18 μm	submicron avg.	supermicron avg.
**Marine Influenced**												
H/C	1.48	1.40	1.44	1.44	1.39	1.44	1.38	1.46	1.39	1.41	1.44	1.41
O/C	0.31	0.32	0.31	0.30	0.31	0.32	0.32	0.33	0.31	0.33	0.31	0.31
% aliph.	36	35	34	38	31	37	34	35	29	46	36	35
% arom.	15	23	20	21	24	19	24	17	20	22	20	21
% lipid-like	30	28	29	29	28	29	27	28	24	26	29	27
% SOA-like	9	9	10	8	7	10	7	9	11	8	9	9
% surf-like	68	64	65	68	68	62	66	62	63	63	66	64
ST min.	36.0	36.8	34.8	39.5	38.9	48.3	37.5	43.9	43.2	41.6	36.8	42.2
**Mixed**												
H/C	1.55	1.55	1.52	1.56	1.49	1.58	1.49	1.55	1.53	1.55	1.55	1.53
O/C	0.27	0.27	0.28	0.27	0.30	0.29	0.30	0.29	0.30	0.31	0.27	0.30
% aliph.	39	38	34	41	33	41	33	38	39	39	38	37
% arom.	11	10	12	9	16	9	16	11	12	10	10	12
% lipid-like	40	36	30	34	27	41	24	32	33	30	36	31
% SOA-like	8	8	9	6	10	7	8	10	8	6	8	8
% surf-like	74	69	69	73	71	72	68	69	69	66	72	69
ST min.	38.5	42.7	40.3	40.0	45.9	37.9	38.3	38.6	41.8	42.3	40.4	40.8
**Continental Influenced**												
H/C	1.53	1.49	1.47	1.48	1.49	1.45	1.50	1.46	1.43	1.47	1.49	1.47
O/C	0.32	0.31	0.37	0.29	0.30	0.32	0.27	0.34	0.34	0.33	0.32	0.32
% aliph.	38	37	33	37	36	29	36	35	29	33	36	33
% arom.	11	13	16	16	15	16	14	15	19	14	14	15
% lipid-like	26	23	16	28	22	21	31	23	17	21	23	23
% SOA-like	12	12	14	8	10	11	6	14	10	9	12	10
% surf-like	64	63	53	67	66	63	73	63	60	66	62	65

The Marine Influenced samples have the largest spreads in formulas, with formulas spanning H/C ranges of 0.25 to 2.5 (Figure 4). Some formulas have high O/C (>0.8) as well, consistent with previous work showing high O/C for organic mass associated with primary marine aerosol (>1.00 for atmospheric primary marine aerosol and >0.50 for mixed marine aerosol) which was not attributed to atmospheric aging.^16^

Different regions of the van Krevelen diagrams and ranges of H/C and O/C can be used to broadly classify organic formulas.^38,96^ Secondary organic aerosol (SOA) has been associated with an H/C range of 1.0 to 1.75 and O/C range of 0.4 to 0.8.^33,38,97^ Here, the SOA region contains ∼9% of the assigned formulas in all three of the samples. The SOA-like organic formulas were slightly more prevalent in the Continental Influenced samples (∼11%), compared to the other two samples (8% for Marine Influenced and 9% for Mixed). Along with the higher concentrations of formate and acetate, this could be indicative of more atmospheric aging in the Continental Influenced samples, compared to the other samples. However, the SOA-like formulas were a small fraction of the total formulas and were similar across the three sampled air mass source regions. SOA has been observed to be a large fraction of the total organic mass in the southeastern United States in the summer.^98,99^ In this study, large organic molecules were concentrated and extracted for characterization using solid-phase extraction, which may exclude smaller SOA molecules. Additionally, some SOA may have formulas that fall outside of the defined O/C and H/C region, which was defined based on laboratory studies.^38,97^

The percentage of molecular formulas that were identified as aliphatic and aromatic compounds varied for the three air mass regions. Aromatic formulas were identified as those with a calculated aromaticity index value of greater than 0.5.^38,58,67,100,101^ In total across all three samples, almost 17% of the organic formulas were classified as aromatic (Table 1). The organic formulas from the Marine Influenced sample had the highest average percentage of aromatic compounds (20%) out of the three samples (Table 1), which may be due to local and transported ship emissions.^33^ This is demonstrated by the distribution of organic formulas in the van Krevelen diagrams, which shows a higher number of formulas with low H/C for the Marine Influenced particles, compared to the other samples (Figure 4).

Formulas were identified as aliphatic molecules if they contained an H/C ratio of >1.0 and a double bond equivalent (DBE)-to-carbon ratio of <0.3.^67^ Across all three samples, almost 36% of the assigned formulas were classified as aliphatic, with little variability in aliphatic fraction between the three samples, each averaging between 34% and 38% (Table 1). However, there are some minor differences when comparing the fractions of aliphatic formulas in different diameter size ranges. For both the Marine Influenced and the Continental Influenced particles, the 10–18 μm diameter size range had two of the lowest percentages of aliphatic compounds (Table 1), suggesting that there are fewer aliphatic compounds in the larger particles. The overall lowest percentage of aliphatic compounds was in the 1.8–3.2 μm diameter size range of the Continental Influenced sample (Table 1). The other two samples, Marine Influenced and Mixed, had above average percentages of aliphatic formulas in their 1.8–3.2 μm size bins. The differences in aliphatic fractions across the size bins for the different sample types are consistent with a different organic mass composition in the Continental Influenced samples, compared to the other samples.

For the samples from all three air mass source regions, the average percentage of aliphatic formulas is higher in the submicron particles than in the supermicron particles, with the Continental Influenced sample having the biggest difference (36%, *n* = 4; and 33%, *n* = 6, respectively), as shown in Table 1. The opposite trend is observed for aromatic assigned formulas, with a slightly higher percentage of aromatic organic compounds in the supermicron particles than in the submicron particles for all samples. The mass spectral characterization shows differences in the organic composition of identified chemical formulas across different diameter size ranges and particle source regions.

### 3.4 Surfactant Fraction of Organic Mass in Particles

#### 3.4.1 Surfactants and Lipid-Like Organic Molecules in Particles

The presence of surfactant molecules in the organic fraction extracted from the particles was confirmed through surface tension measurements. The surface tension minimums (surface tensions of the organic extracts rehydrated in 40 μL of ultrapure water) are shown in Table 1 for the Marine Influenced sample and the Mixed sample. The minimum surface tensions are significantly depressed compared to a surface tension of pure water (72 mN m^–1^), across all of the particle size bins and in both sample types (*n* = 20), indicating the presence of surfactants.

The minimum surface tension is significantly lower for submicron particles than supermicron particles (unpaired Student’s *t*-test, *p* < 0.05), with mean values of 36.8 mN m^–1^ (*n* = 4) and 42.2 mN m^–1^ (*n* = 6), respectively, in the Marine Influenced sample (see Table 1, as well as Figure S6 in the Supporting Information). This indicates that submicron particles had higher concentrations of surfactants, or stronger surfactants, which had a large effect on the surface tension. This trend was not observed in the Mixed sample where the surface tension minimums were similar at 40.4 mN m^–1^ (*n* = 4) and 40.8 mN m^–1^ (*n* = 6) for the submicron and supermicron particles, respectively. This trend confirms that the influence of surfactants in the Mixed sample was different from that of the Marine Influenced sample, based on the source region of the air mass.

The van Krevelen diagrams were also used to broadly classify lipid-like formulas,^38,96^ which have previously been identified to be surfactant-like. Lipids generally have formulas with high H/C (>1.5) and low O/C (<0.3) (see Figure 4, as well as Figure S7 in the Supporting Information). Over 27% of the chemical formulas across all three samples and size ranges were identified as lipid-like (n = 30). The highest percentage of lipid-like formulas identified was in the Mixed particles (Table 1). The Continental Influenced samples had the highest percentage, ∼31%, of formulas that had low O/C (<0.3) and lower H/C (<1.5), identified as hydrocarbon-like.

To expand the classification and identify formulas that may be surfactant-like, in addition to the lipid-like formulas, the H/C and O/C of known surfactant standards from a vendor database were calculated (*n* ≈ 2400). A van Krevelen diagram was plotted to visualize the distribution of these surfactant formulas (see Text S2 and Figure S7 in the Supporting Information). More than 73% of the surfactant standards contained H/C ratios >0.4 and O/C ratios <0.35, and this region was thus classified as “surfactant-like” (Figure S7 in the Supporting Information). Previous work has also identified surfactants with similar H/C and O/C formulas.^34,35,58^

**Figure 5:**
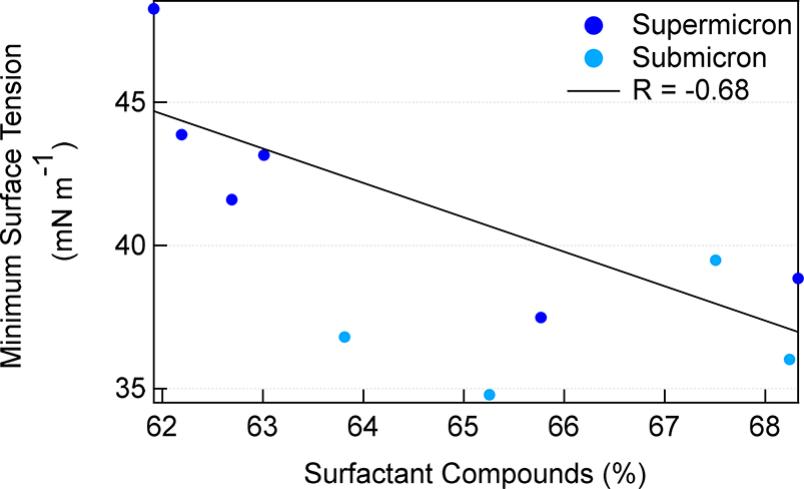
Correlation between the percentage of organic formulas with H/C and O/C values in the surfactant-like range and the associated surface tension minimums for the Marine Influenced samples (*n* = 10). Markers are colored by supermicron (dark blue) or submicron (light blue) particle size ranges.

Using this classification for surfactant-like organic molecules, more than 60% of formulas identified in the Marine Influenced samples were classified as surfactant-like. A negative correlation (*R* = −0.68, *n* = 10) was observed between the percentage of formulas in the surfactant region and the minimum surface tension of the Marine Influenced samples for the different particle size ranges (Figure 5). This negative correlation demonstrates that this mass spectral classification identified the surfactant-like formulas that have the most influence on the surface tension of the Marine Influenced samples. Here, it is generally observed that the organic extracts from the submicron particles have a larger fraction of surfactant-like compounds and a lower minimum surface tension than those from the supermicron particles (Table 1, Figure 5). This is also consistent with previous work that observed a larger enrichment of surfactant fluorescent organic matter in generated sea spray aerosol as the particle size decreased.^102^ Previous work on marine aerosol observed a larger fraction of water-soluble organic compounds in fine particles, compared to coarse particles, and saw that the average surface tension of the particle extracts decreased as a function of water-soluble organic carbon concentration, inferring that the aliphatic and humic-like composition would result in surface-active properties.^8,103^ A correlation of this strength is not observed with the Mixed samples (see Figure S8 in the Supporting Information, *n* = 10), indicating that the identified formulas may be more variable in composition and strength in the Mixed sample across the different size ranges.

#### 3.4.2 Fatty Acid Chain Length Varies with Air Mass Source Region

Sources of surfactants can differ for each sample air mass region. The Marine Influenced particles likely had surfactants from seawater that aggregate toward the sea surface and are aerosolized in the wave breaking and bubble bursting process. Seawater can be a source of different types of surfactants, including fatty acids, which have previously been characterized in particles produced by replicating the open ocean bubble bursting process.^35^ Fatty acids can also be emitted from terrestrial sources, such as trees and other vegetation,^104^ and may contribute significantly to surfactant concentrations in particles.^40^ Fatty acids are also emitted during biomass burning and during coal burning.^37,105^

Chemical formulas from high-resolution mass spectrometry analyses of the samples from the three source regions were compared to a list of identified molecules from Cochran et al.^35^ that contained saturated and unsaturated fatty acids, saturated oxo-fatty acids, saturated hydroxyl-fatty acids, saturated dicarboxylic acids, sulfates, and linear alkylbenzenesulfonates. In the particles from the three source regions, the most commonly observed molecules were tetradecanedioic, pentadecadienoic, and oxooctadecanoic acid. The unsaturated fatty acids, such as pentadecadienoic acid, were mainly found in the Mixed and Continental Influenced samples and were often found in both submicron and supermicron particles. Unsaturated fatty acids are often associated with short lifetimes due to the reactivity of the molecules,^106,107^ so this indicates that these fatty acids were likely from nearby terrestrial or anthropogenic sources.

**Figure 6:**
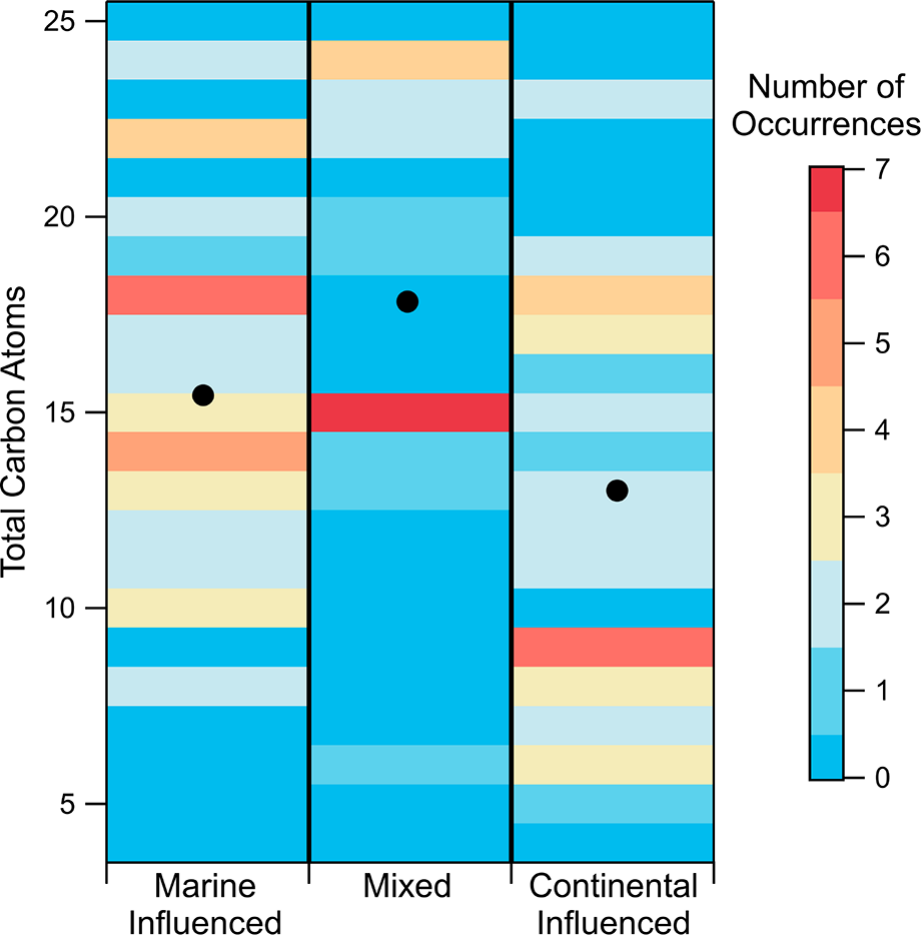
Number of occurrences of an identified fatty acid formula containing a specific number of carbon atoms for each sample. The average number of carbons for each sample is shown as a black marker.

The distribution of the identified fatty acid formulas in each sample, as a function of the number of carbon atoms in the formulas, is shown in Figure 6. The fatty acids from the Marine Influenced particles (*n* = 10) contained an average of 15.8 carbons, which is consistent with previous studies of fatty acids in marine aerosol particles.^35,40,43^ The Mixed samples (*n* = 10) had an average of 18.2 carbons per formula, and the Continental Influenced samples (*n* = 10) had 13.0. The low average number of carbons in fatty acids for the Continental Influenced samples is unexpected for biomass burning or fossil fuel producing particles and is likely mainly driven by other anthropogenic influences.^40^

Additionally, the most abundant fatty acid carbon chain lengths were compared for the three different samples (Figure 6). The most abundant fatty acid carbon chain length is 18 for the Marine Influenced sample, 15 for the Mixed sample, and 9 for the Continental Influenced sample. This further suggests that the Marine Influenced particles may contain fatty acids that were recently emitted from a marine source while the Continental Influenced particles may contain fatty acids from different sources, such as from plant waxes, or those that were fragmented due to photochemical aging in the atmosphere.^40,108−112^

## 4.0 Conclusion

This is one of the first studies to measure the organic composition of surfactant-like molecules in size-resolved atmospheric aerosol particles using high-resolution mass spectrometry. Large organic compounds were extracted from the particles collected in 10 size ranges by using solid-phase extraction. The concentrations of the major ions in particles combined with HYSPLIT analyses were used to identify samples originating from three distinct air mass source regions: Marine Influenced, Mixed, and Continental Influenced. The composition of the particles, including the major ions and organic fraction, varied with the air mass source region and particle size ranges.

Surfactant-like organic compounds were observed across all particle size ranges and in particles from all three source regions. Surface tension minimums, measured for the organic extracts, were significantly depressed (<45 mN m^–1^, *n* = 20) in all measured samples, confirming the presence of surfactant-like molecules. For the Marine Influenced sample, the surface tension minimum was lower in the submicron particles, compared to the supermicron particles, indicating stronger surfactants or higher concentrations of surfactants in the submicron Marine Influenced particles.

The van Krevelen diagrams show the formulas of many of the extracted organic compounds to be consistent with surfactants, with high H/C and low O/C values across all size bins and air mass source regions. Generally, the formulas assigned for organic molecules in particles in the submicron size range had higher H/C values than those in the supermicron particles for all sample sets. The percent aliphatic and percent aromatic were similar across size ranges for given sampling times. For the Marine Influenced particles, the fraction of formulas that fell in the H/C and O/C regions identified as surfactant-like had a negative correlation with surface tension minimums and comprised 61% of the total assigned formulas.

Formulas identified as fatty acids were observed in particles from all three of the source regions. The Marine Influenced sample had the highest abundance of longer chain fatty acids (C18), and the Continental Influenced sample had the highest abundance of shorter chain fatty acids (C9). This may indicate that the organic compounds in the Continental Influenced particles originated from different sources or were aged in the atmosphere through fragmentation of larger organic molecules compared to the organic compounds in the Marine Influenced particles. All measurements completed in this work confirm three distinct populations of particles exhibiting different compositions and relationships to surfactants found in the extracted organic fractions.

This exploratory work can be expanded to further investigate the influence of a larger range of particle sources on the composition of surfactants in atmospheric particles and to identify correlations between surface tension minimums and fraction of surfactant-like formulas across a wider range of particle sources and within a given size range. Ongoing work to measure the surfactant concentrations across the particle size ranges and comparison of the surfactant properties and formulas to the total concentrations are essential to broadening the understanding of the sources of this class of molecules and their role in particle formation and physiochemical characteristics.

## Supporting Information Available

Table showing the concentration of inorganic ions in each size bin measured using ion chromatography (Table S1); text explaining the scaling of the Continental Influenced APS number distributions (Text S1); figure showing the HYSPLIT back trajectories for each of the first 6 days (Figure S1); figure showing the HYSPLIT back trajectories for each of the last 7 days (Figure S2); figure showing the HYSPLIT back trajectories for the sample identified as from the Marine Influenced air mass source region (Figure S3); figure showing the HYSPLIT back trajectories for the sample identified as from the Mixed air mass source region (Figure S4); figure showing the HYSPLIT back trajectories for the sample identified as from the Continental Influenced air mass source region (Figure S5); figure showing the surface tension at each particle size for the Marine Influenced sample (Figure S6); text explaining the use of a vendor database to create a van Krevelen for surfactant standards (Text S2); figure showing the van Krevelen diagram for surfactant standards (Figure S7); figure showing correlation between surfactant-like formula percentage and surface tension for the Mixed samples (Figure S8) (PDF)
