# Supplementary material for: Influence of Air Mass Source Regions on Signatures of Surface-Active Organic Molecules in Size Resolved Atmospheric Aerosol Particles
Source: ACS Earth Space Chem. 2023 Aug 2;7(8):1578–91. doi: 10.1021/acsearthspacechem.3c00161 (PMC10441572; doi:10.1021/acsearthspacechem.3c00161)
Supplement: Supplementary file 1 — sp3c00161_si_001.pdf [file sp3c00161_si_001.pdf]

Supporting Information for

Influence of Air Mass Source Regions on Signatures of Surface-Active Organic Molecules in Size Resolved Atmospheric Aerosol Particles

Tret C. Burdette[1], Rachel L. Bramblett[1], Kathryn Zimmermann[2], Amanda A. Frossard[1,*]

1. Department of Chemistry, University of Georgia, Athens, GA, 30606, USA.
2. Department of Chemistry, Georgia Gwinnett College Lawrenceville, GA, 30043, USA.

*Corresponding author: Amanda A. Frossard, afrossard@uga.edu

Number of pages: 11

Number of tables: 1

Number of figures: 8

Text S1: The particle number size distributions of the Scanning Electrical Mobility Spectrometer (SEMS) and Aerodynamic Particle Sizer (APS) overlapped well at the merge diameter for the Marine Influenced and Mixed samples. For the Continental Influenced sample, the APS number size distributions were roughly an order of magnitude lower than the SEMS number size distributions at the merge diameter. This was determined to be a sampling issue with the APS measurements, and the APS number size distributions were multiplied by a factor of 10.8 to match the SEMS number size distributions at the merge diameter. For all three samples, using a particle density of 2.12 g cm^{-1} to convert SEMS mobility diameters to aerodynamic diameters gave the best overlap between the SEMS and APS number size distributions at the merge point.

Table S1: Concentrations (µg m^{-3}) of major ions and total concentrations in each size bin of each sample. Ion concentrations listed as 0 are below detection limit.

	**Marine Influenced**									
Ion	<0.18 µm	0.18-0.32 µm	0.32-0.56 µm	0.56-1.0 µm	1.0-1.8 µm	1.8-3.2 µm	3.2-5.6 µm	5.6-10 µm	10-18 µm	>18µm
Lithium	0	0.0002	0.0004	0	0.0003	0.0007	0.0004	0.0005	0.0005	0.0006
Sodium	0	0.1216	0.3131	0	0.0426	0.7475	0.9264	0.4855	0.4100	0.2869
Ammonium	0	0	0	0.0018	0	0	0.0022	0	0	0
Potassium	0	0	0	0	0	0	0	0	0	0
Magnesium	0	0.0013	0	0	0	0.0001	0	0.0020	0	0.0037
Calcium	0.0476	0.0461	0.0276	0.0341	0.0071	0.0245	0.0169	0	0	0
Floride	0	0	0.0006	0	0.0001	0	0.0002	0	0.0004	0.0001
Acetate	0	0	0	0	0	0	0	0	0	0
Formate	0	0	0	0	0	0	0	0	0	0
Chloride	0	0.0390	0.0919	0	0.0931	0.4221	0.9147	0.2690	0.1531	0.0881
Nitrite	0	0	0	0	0	0.0037	0.0042	0	0	0
Carbonate	0	0.3034	0.2314	0	0	0.2971	0.2141	0.2997	0.0664	0.0506
Sulfate	0	0	0	0	0	0	0	0	0	0
Nitrate	0	0	0	0	0	0.0245	0.0390	0	0	0
Total	0.047	0.511	0.6651	0.0359	0.1433	1.5205	2.1185	1.0567	0.6307	0.4302
	**Mixed Influenced**									
Lithium	0	0.0007	0.0010	0.0002	0.0009	0.0005	0.0009	0.0007	0.0006	0.0009
Sodium	0	0.4398	0.8951	0.0559	0.6621	0.7124	1.1745	0.7478	0.4756	0.3138
Ammonium	0	0.0194	0.0007	0	0.0267	0.0052	0.0172	0.0713	0.0003	0.0004
Potassium	0	0.0200	0.0404	0.0049	0.0202	0.0269	0.0426	0.0438	0.0045	0.0266
Magnesium	0	0.0005	0	0	0	0	0.0054	0.0030	0	0
Calcium	0	0.0134	0.0041	0.0173	0	0	0.0321	0.0845	0.0056	0.0083
Floride	0	0.0009	0.0002	0	0.0013	0.0025	0.0019	0.0070	0.0009	0.0006
Acetate	0	0.0418	0	0	0.0467	0.1256	0.1128	0.3230	0.0654	0.0455
Formate	0	0.0159	0.0190	0	0.0174	0.0264	0.0433	0.0952	0.0319	0.0041
Chloride	0	0.0319	0.0526	0.0222	0.2957	0.5459	1.1807	0.5554	0.1665	0.0706
Nitrite	0	0	0	0	0.0022	0.0115	0.0050	0.0140	0.0023	0
Carbonate	0.6113	0.8542	0.8512	0.2672	0.5825	0.4491	0.8863	0	0.1940	0.2057
Sulfate	0	0	0.0784	0	0	0.0011	0.0044	0	0	0
Nitrate	0.0373	0	0.0073	0.0048	0.0153	0.0510	0.1231	0	0	0.0063
Total	0.6487	1.4383	1.9501	0.3726	1.6711	1.9583	3.6303	1.9458	0.9475	0.6829
	**Continental Influenced**									
Lithium	0.0003	0	0.0002	0	0.0001	0.0001	0	0.0001	0	0
Sodium	0.8858	0.2603	0.4710	0.0905	0.0209	0.0406	0.1839	0.3533	0.1925	0.2193
Ammonium	0.0450	0.0214	0.1056	0.0753	0	0	0.0256	0	0	0
Potassium	0.0327	0.0126	0.0618	0.0169	0	0	0.0077	0.0165	0.0050	0
Magnesium	0.0194	0.0029	0.0043	0.0075	0.0012	0.0005	0.0058	0.0006	0	0
Calcium	0.0326	0.0381	0.0267	0.0384	0	0	0.0105	0.0224	0	0
Floride	0.0011	0.0007	0.0013	0.0007	0.0002	0	0	0.0002	0	0
Acetate	0.3704	0	0	0.2448	0	0	0.2272	0	0	0
Formate	0.0914	0.0040	0	0	0	0	0.0699	0.0403	0.0199	0
Chloride	0.8223	0.0356	0.0014	0.0081	0.0054	0.0145	0.0141	0.0513	0.0028	0.0424
Nitrite	0.0022	0.0000	0.0005	0.0017	0	0	0	0	0	0
Carbonate	0.3367	0.3571	0.7568	0.2139	0.0803	0.1040	0.1518	0.3692	0.2934	0.1711
Sulfate	0.0376	0.0418	0.0122	0.0162	0	0	0.0238	0.0157	0.0014	0.0100
Nitrate	0.0076	0	0.0149	0	0	0	0	0.0001	0.0019	0
Total	2.6850	0.7744	1.4567	0.7140	0.1080	0.1597	0.7204	0.8698	0.5169	0.4428

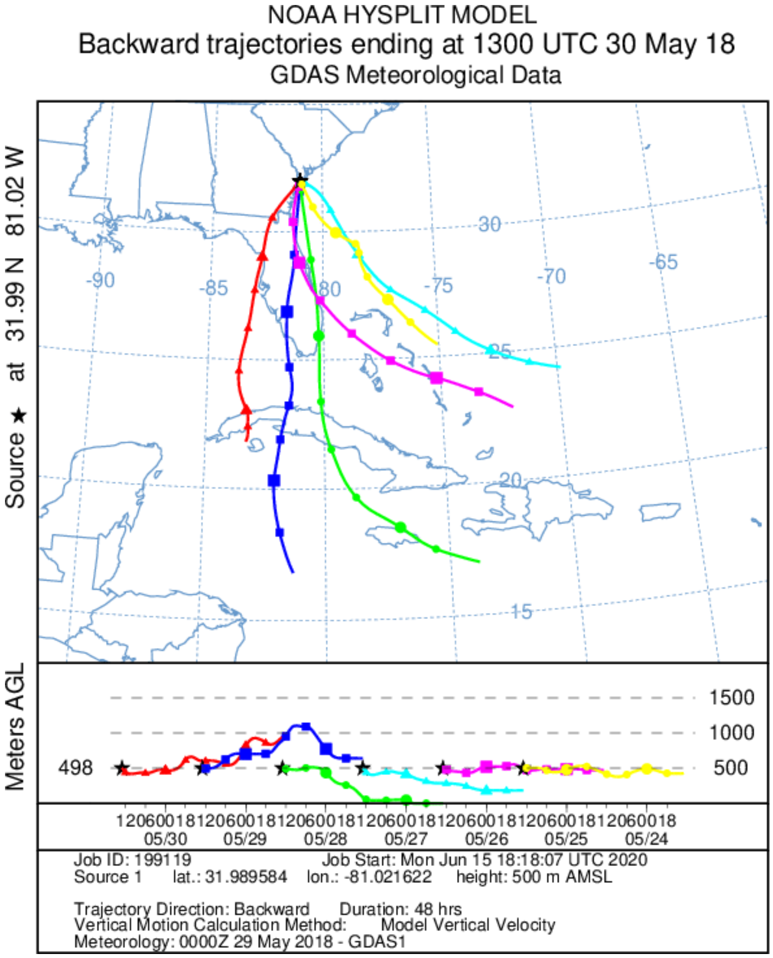

Figure S1: Hybrid Single-Particle Lagrangian Integrated Trajectory (HYSPLIT) 48-hour back trajectories for the first 6 days of the project sampling period (May 25 to May 30, 2018).

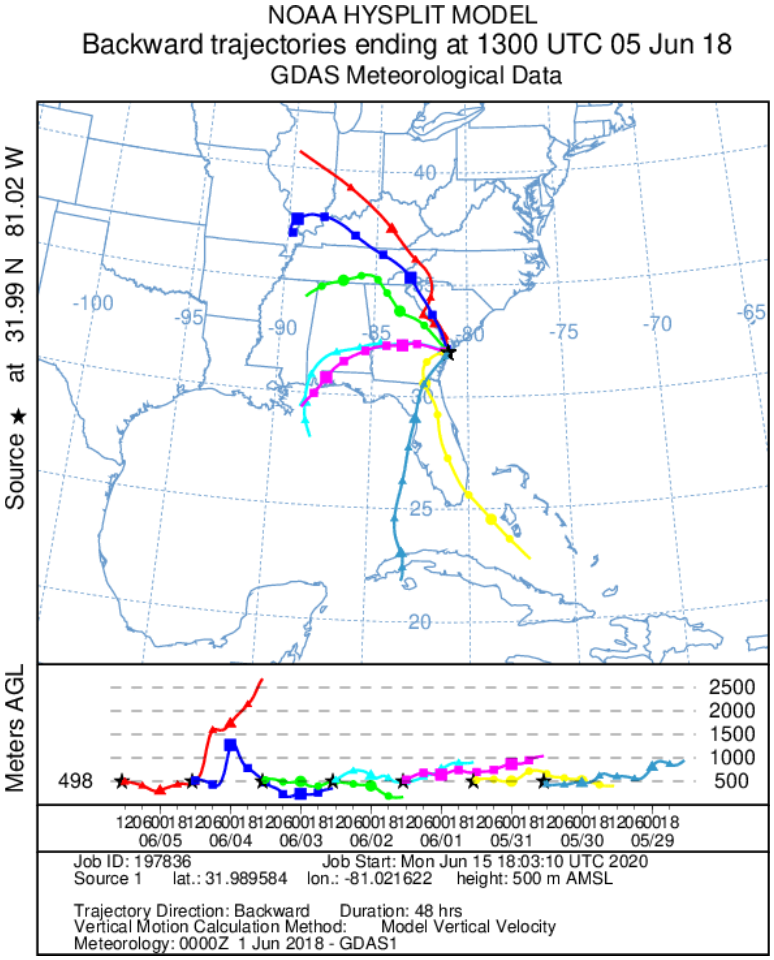

Figure S2: HYSPLIT 48-hour back trajectories for the final 7 days of the project sampling period (May 30 to June 5, 2018).

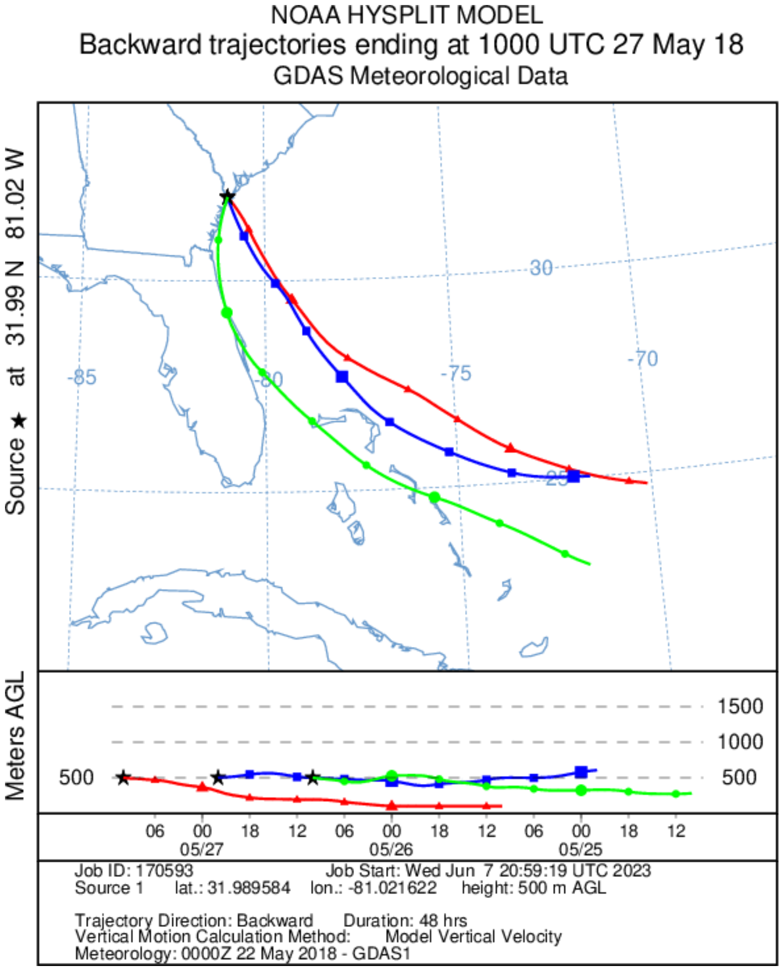

Figure S3: HYSPLIT 48-hour back trajectories calculated for the May 26-27 (Marine Influenced) sample period.

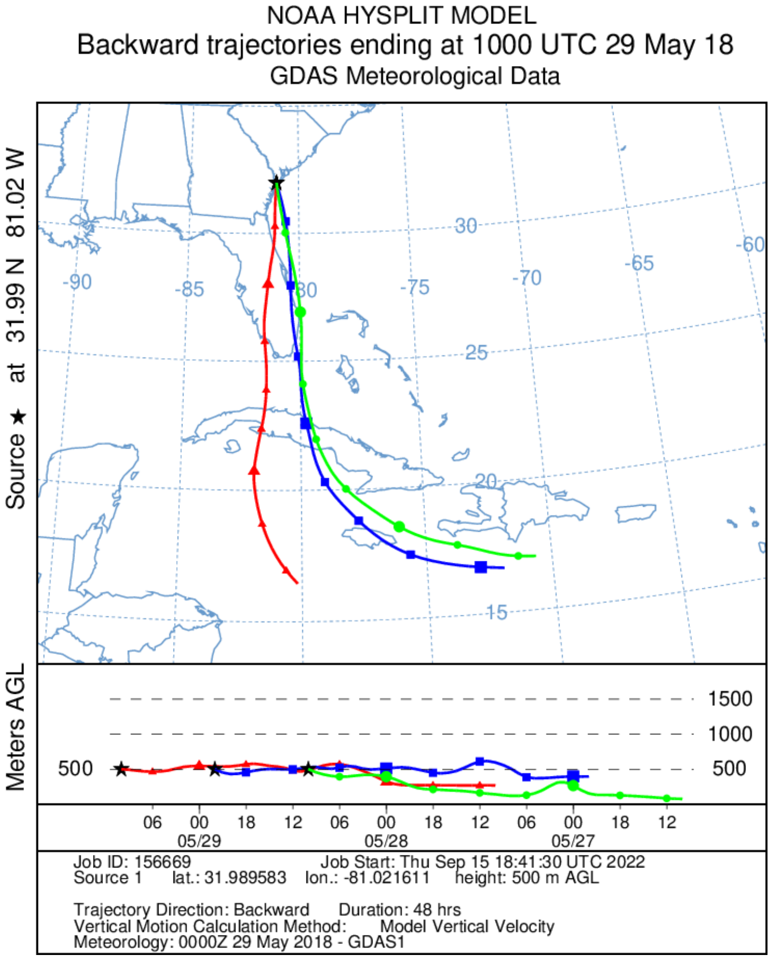

Figure S4: HYSPLIT 48-hour back trajectories calculated for the May 28-29 (Mixed) sample period.

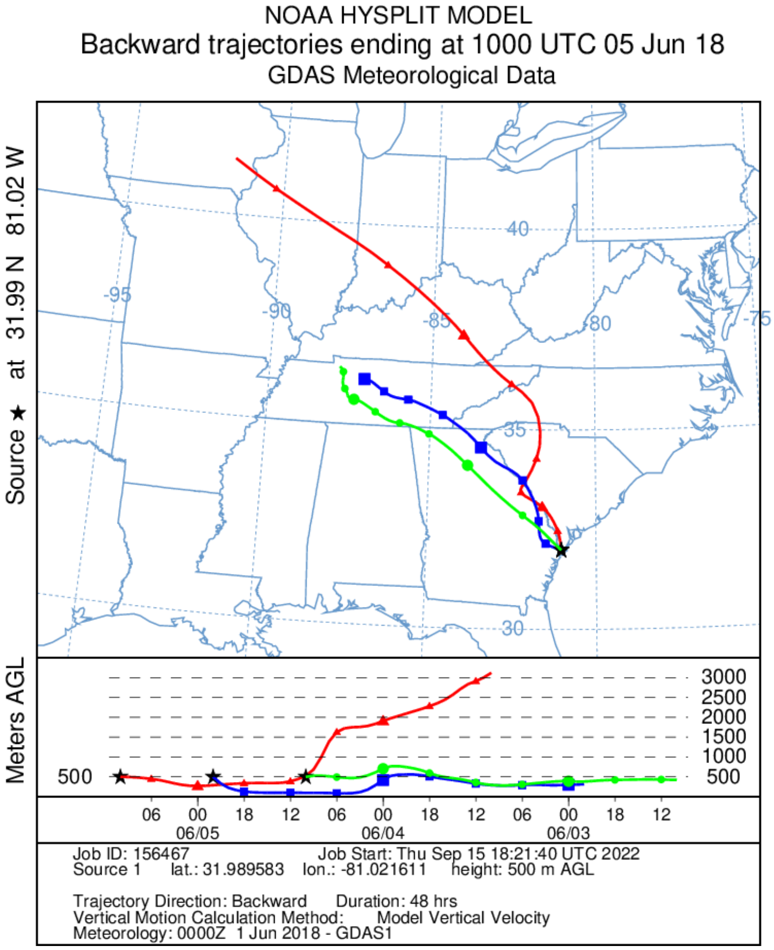

Figure S5: HYSPLIT 48-hour back trajectories calculated for the June 4-5 (Continental Influenced) sample period.

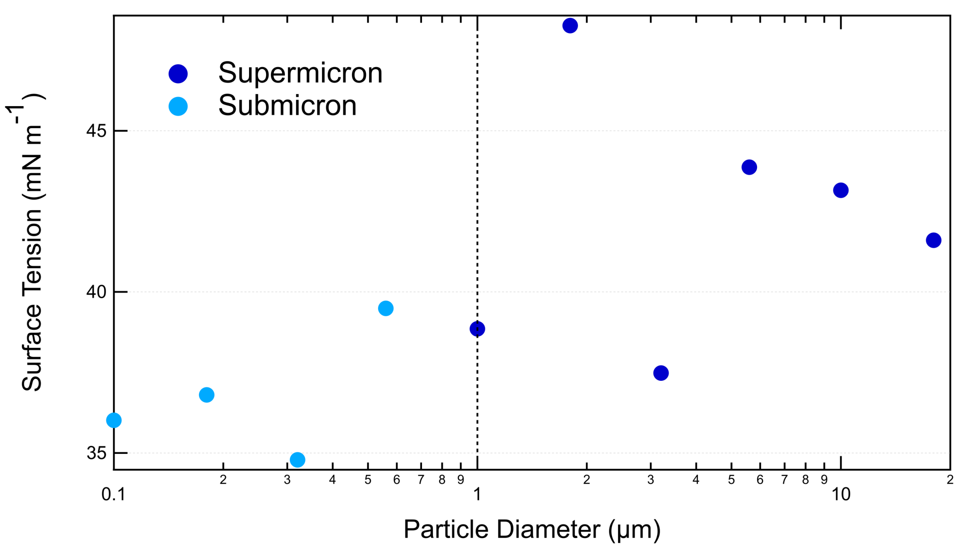

Figure S6: Surface tension minimums for the Marine Influenced aerosol particle samples. Markers are colored based on particle size for submicron (light blue) and supermicron (dark blue) diameter size ranges.

Text S2: Formulas of surfactant standards, both natural and synthetic, were used to identify a region of the van Krevelen diagram that contained a majority of the surfactants. This was done using a list of surfactants and formulas from the website of the surfactant chemical vendor Alfa Chemistry. They have approximately 2400 surfactants listed (~ 600 anionic, ~200 cationic, and ~1600 nonionic) and a majority have listed chemical formulas. Ratios of hydrogen to carbon (H/C) and oxygen to carbon (O/C) were calculated for each formula and plotted in a van Krevelen diagram (Figure S7).

The region of H/C > 0.4 and O/C < 0.35 (outlined in black in Figure S7) contains more than 73% of the surfactant formulas from the known standards. This region is classified as surfactant-like, since it contains a large fraction of the known surfactant formulas and is consistent with formulas that may have a surfactant-like structure, such as lipids, fatty acids, aliphatic groups, etc. The low H/C of some of the formulas is a result of nitrogen or sulfur present in the known surfactant formulas.

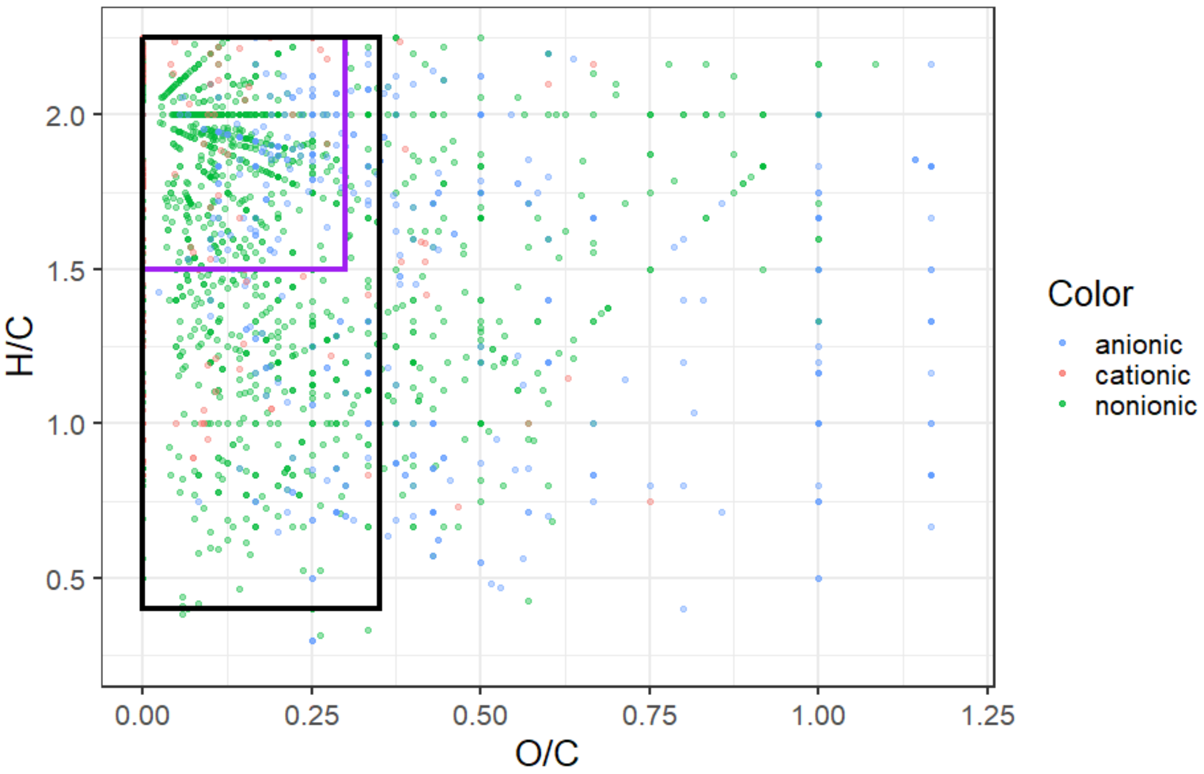

Figure S7: The van Krevelen diagram calculated from standard surfactant molecular formulas from Alfa Chemistry. The purple region indicates lipid-like compounds, and the black region indicates surfactant-like formulas. Over 73% of surfactant standards fell within the black region of the van Krevelen diagram.

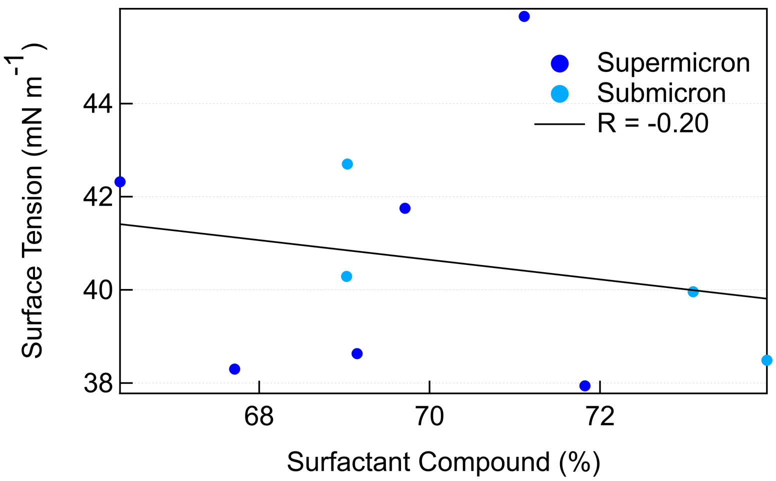

Figure S8: Correlation between the percentage of organic formulas with H/C and O/C values in the surfactant-like range and the associated surface tension minimums for the Mixed samples (n = 10). Markers are colored by supermicron (dark blue) or submicron (light blue) particle size ranges.
